# The Swindon Foot and Ankle Questionnaire: Is a Picture Worth a Thousand Words?

**DOI:** 10.5402/2012/105479

**Published:** 2012-09-26

**Authors:** Rosemary Waller, Peter Manuel, Lyn Williamson

**Affiliations:** Department of Rheumatology, Great Western NHS Foundation Trust, Swindon SN3 6BB, UK

## Abstract

*Objectives*. Despite increased awareness of the high prevalence and significance of foot and ankle problems in rheumatoid arthritis (RA), feet remain neglected. Reasons may include the perception that feet are difficult to assess, they are not included in the DAS28, and lack of freely available foot screening tools specific for RA. *Methods*. The Swindon Foot and Ankle Questionnaire (SFAQ) is a simply worded 10-point foot and ankle screening questionnaire with diagrams of feet and ankles for use in general rheumatology outpatients. All RA patients on our electronic database were invited to complete the questionnaire and attend clinic for assessment. Patients assessed clinically were scored out of 10 using the parameters from the questionnaire. The SFAQ was compared to the Manchester Foot Pain and Disability Index (MFPDI), DAS28, HAQ, HAD, and OSRA scores. *Results*. 597 questionnaires were sent, 301 (50%) returned, and 137 seen in clinic. There was good correlation between the postal SFAQ score, clinic score (*r* = 0.63), and the MFPDI (*r* = 0.65). Neither of the foot scores correlated with other RA disease outcome measures. 75% patients completed the picture. 73% corresponded to clinical findings. 45% of patients required an intervention following clinical review and trended towards higher scores. *Conclusions*. The SFAQ was quick to complete and correlated with the MFPDI. Lack of association with standard RA outcome measures suggests that relying on these scores alone may miss foot pathology. The diagrams were a useful complement. This simple screening tool could aid identification of RA foot and ankle problems.

## 1. Introduction

Foot and ankle problems affect 90% of rheumatoid arthritis (RA) patients [1, 2] and can be important factors in reducing quality of life, disability, and depression [3–8]. Recent national guidelines emphasise the importance of podiatric assessment both at the time of diagnosis and as part of ongoing routine care [9–12]. Despite this feet remain a neglected area. Contributory factors include the perception that foot assessment is complicated and time consuming, with medical staff having varying experience and confidence, and patients' difficulty in accurately describing foot and ankle problems [13]. The widespread use of the DAS28 score, which does not include the feet and ankles, means that clinically important problems may be missed. 

A recent survey of our patient population to establish the prevalence of foot problems highlighted the need for a rapid screening tool for use in clinic to yield useful practical information to determine which patients were suffering from symptomatic foot pathology [14]. Currently available screening tools for foot problems are either not specific to RA, did not include the ankle, are lengthy, are not freely available, or were not freely available to use if developing your own foot score. 

**Figure 1:**
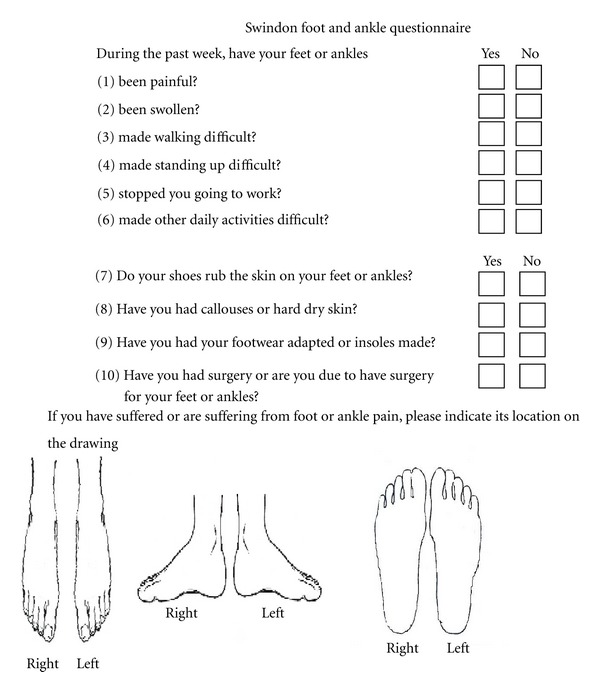


We present the Swindon Foot and Ankle Questionnaire (SFAQ) (Figure 1). This consists of ten short questions with binary answers plus diagrams of the feet. Patients should be able to complete the questionnaire unaided in less than one minute. The scoring process should take a few seconds.

The brevity was to minimise the problems on questionnaire fatigue.

## 2. Methods

### 2.1. Development of Swindon Foot and Ankle Questionnaire 

#### 2.1.1. Language, Literacy, and Legibility

The questionnaire was designed to be easy to read to improve completion rates by patients. 16% of adults in the UK are functionally illiterate, defined as a reading age at or below that of an 11 year old [15, 16]. The readability of any piece of written information can be assessed using a variety of validated formulas, with the SMOG formula thought to be most relevant for medical literature [17].

The unique feature of our questionnaire is the inclusion of diagrams of feet and ankles to help those completing the questionnaire with literacy problems, those who have visual rather than verbal understanding, those for whom English is not their first language, and those short of time. We anticipated that the use of the diagram would be particularly useful in patients with multiple areas of foot pathology, and for areas such as the hindfoot, where confusion occurs between ankle and subtalar arthritis and tendinitis.

#### 2.1.2. Questions

We chose to use 10 questions to ensure that total scores were easy to calculate. The questions have binary “yes”/“no” answers for ease of patient understanding and to simplify scoring. We included both foot and ankle, as the hindfoot is a common area of symptoms in RA, and is not included in other foot screening tools. The questionnaire assesses foot and ankle problems affecting the following four domains: symptoms (questions 1 and 2), function (questions 3–6), disability (questions 7-8), and previous interventions (questions 9-10). 2 questions were posed for each domain to minimise redundancy of questions. Each domain was weighted 2, with the exception of function which was weighted 4. The question about work was included as a “yellow flag” to prompt urgent assessment and referral if necessary.

The questions were chosen by group discussion with members of the rheumatology and podiatry department. The wording of the questions was further modified following the results of an anonymous postal pilot of 448 patients by further group discussion [14]. A question asking patients whether they felt that they needed intervention for their feet was felt to assess patient education rather than symptoms and was replaced by asking about callouses.

### 2.2. Assessment of SFAQ Validity

We invited all 597 RA patients (as defined by a consultant rheumatologist) on our electronic database to complete the SFAQ. Postal questionnaires returned were scored out of 10, with one point being given to each yes answer and no scoring zero. The picture of foot and ankle was assessed after clinical review of the patient and assigned as “not completed,” “accurate,” or “not accurate” as matched to clinical assessment.

**Figure 2:**
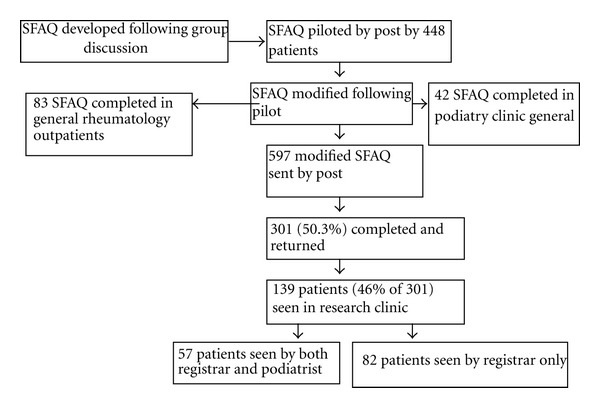


All patients completing the SFAQ by post were invited to attend a research clinic appointment to have their feet and ankles assessed as part of a general health screen (Figure 2). 

Patients were seen in clinic between 2 and 8 months after originally completing the SFAQ by post. The assessment at this appointment was undertaken by the rheumatology registrar (RW). At this clinic appointment, patients completed a Manchester Foot Pain and Disability Index (MFPDI), Health Assessment Questionnaire (HAQ) and Hospital Anxiety and Depression Score (HADS) questionnaire [18–20]. A foot and ankle history and examination were undertaken together with a general rheumatology examination. DAS 28 and overall status in rheumatoid arthritis (OSRA) scores were calculated [21, 22].

 A random sample of these patients also had an independent consultation carried out by an experienced podiatric surgeon (PM), who was blinded to the postal questionnaire result and the assessment of RW. The parameters of the questionnaire were assessed clinically and a score out of 10 calculated as above. 

Any intervention carried out at this clinic visit or arranged as a result of it was recorded. Interventions included joint injection, fitting of orthotic devices and referral, to surgery. We hoped to determine whether there was a sensitive and specific threshold to predict likelihood of requiring intervention from the total score from the SFAQ.

We tested the validity of the questionnaire using Tugwell and Bombadier's methodological framework as our guide [23]. They described 5 types of validity (face, content, construct, criterion, and discriminant) that need to be fulfilled when evaluating screening tools used in the assessment of RA. 

Face validity was assessed by distributing the SFAQ to general rheumatology outpatient clinics. Here it was completed by all patients attending on a single day to assess its usability. The 3 consultants seeing these patients were asked to comment on the usefulness and length of time it took to complete and discuss the questionnaire. 

The SFAQ was also completed by patients attending a general podiatry clinic. At this visit patients also completed a 10 cm foot pain visual analogue score (VAS) to assess its construct validity.

Construct and criterion were evaluated by comparison with the MFPDI, OSRA, HAQ, and HAD. Reproducibility of the questionnaire was assessed by comparison of the scores given by RW and PM. 

Ethical approval was obtained from Wiltshire Research Ethic Committee and written consent obtained from all patients. Microsoft Access 2007 was used to perform statistical analysis. All correlation coefficients were calculated as Spearman rank correlation coefficients.

## 3. Results

Of 597 questionnaires sent, 301 (50.3%) were returned (Figure 2). 139 patients (from the 301 questionnaire returners) were seen in research clinic by RW, with 57 of the 139 also seen by PM. 

Baseline demographics of the patients returning questionnaires are as follows: 69% female with a mean age of 62 (range 31–91). Mean duration of RA was 12 years (range 1–40 years). There were no demographic differences between the patients assessed in clinic compared with those who only returned a postal questionnaire. 

Clinical scores are shown in Table 1. 

16 patients (11%) rated their SFAQ score as 0. Of the 139 patients seen in the research clinic, 65 (46%) required intervention with 9 (7%) requiring more than 1 (Table 2). 

A wide variety of pathologies were diagnosed, with 92% of patients assessed at research clinic having some form of foot or ankle pathology documented and 66% having more than one problem (Table 3). 43% of patients had forefoot pathology, 31% hindfoot pathology, and 21% nonarticular pathology.

A further 83 patients with inflammatory arthritis completed questionnaires during general rheumatology outpatients. Their median SFAQ score was 3 (range 0–9). 65 of these patients (78%) had their feet examined. The pathologies identified are outlined in Table 4. 12 patients (18%) had more than one foot pathology identified. 

In addition 42 patients with inflammatory arthritis completed questionnaires in general podiatric clinic. The median foot score was 5 (range 1–10). A broad range of diagnoses were again identified, with 18 patients (43%) having more than one pathology (Table 3). 39 (93%) of these patients required intervention (Table 4).

In total (including postal returns, rheumatology, and podiatry clinics), 426 questionnaires were completed, with 264 completed in a clinical setting (research, rheumatology, and podiatry clinic).

## 4. Face Validity: Credibility. Is the Measure Sensible?

The SFAQ has been used by 3 rheumatology consultants, 2 rheumatology registrars, 2 rheumatology nurse practitioners, and 2 podiatric surgeons. It was felt that the SFAQ was easy to use and that sensible questions were asked. Of the 38 questionnaires returned from general rheumatology clinics with an indication of its usefulness, 34 respondents felt that it had been a useful adjunct. No one felt that completing the SFAQ added to the length of consultation. 

## 5. Content Validity: Comprehensiveness. Does the Measure Sample Multiple Domains of RA Foot and Ankle Problems?

The five domains of symptoms (questions 1 and 2), function (questions 3–6), disability (questions 7-8), and previous interventions including footwear adaptation and surgery (questions 9-10) affecting both the foot and ankle were compared. The use of the diagram was assessed.

 There was a moderate correlation between the symptom and function domains, with *r* = 0.65. There was poor correlation between the other domains of disability and interventions, and symptoms and function did not correlate with these domains either. This suggests that each dimension is relatively independent and that duplication is not occurring. 

Of 139 research clinic patients, 72% completed the diagrams. 90% of patients with an SFAQ of 4 or greater completed the diagram, with 71% (55/77) having done so accurately enough that a diagnosis could be made using it. Those who completed the diagrams had a tendency towards a higher foot score, particularly if the diagram was accurate. As expected patients with no foot problems did not complete the diagram. 59 (71%) patients from general rheumatology clinic completed the diagrams, of whom 42 (71%) were felt to have done so accurately. 

## 6. Construct Validity: Does the Measure Make Biological Sense and Do the Results Agree with Other Measures Which Claim to Measure the Same Thing?

The SFAQ was compared with the MFPDI in research clinic and the 10 cm foot pain VAS in general podiatry clinic. The MFPDI has been previously validated in patients attending a general rheumatology clinic and so was felt to be a useful comparator to the SFAQ.

The SFAQ correlated with the MFPDI, with *r* = 0.65. 

There was poor correlation between the VAS and the SFAQ score—*r* = 0.36. 

## 7. Criterion Validity: Does the Measure Predict or Correlate with Standard Measures of RA Outcome? 

The SFAQ was compared with DAS28, OSRA activity and damage scores, HAQ, and HAD.

The SFAQ did not correlate with any of these disease outcome measures with *r* ranging from 0.18–0.29. The MFPDI similarly did not correlate with any of these scores either (*r* range 0.25–0.33). 

The OSRA activity score and DAS 28 correlated with each other (*r* = 0.59). Neither the OSRA activity or DAS 28 scores in our patients correlated with damage and disability outcome scores such as the OSRA damage score, HAQ or HAD. 

## 8. Discriminant Validity: Sensitivity to Change. Does the Measure Detect the Smallest Clinically Important Change?

Intervention rates were compared with the SFAQ score in order to determine whether there was a sensitive and specific threshold to predict likelihood of requiring intervention. 

As the SFAQ score increased, the specificity of determining who required intervention rose but the sensitivity fell (Table 5). A threshold of a score of 4 or greater appeared to have the best positive and negative predictive values of 0.69 and 0.54, respectively.

## 9. Reproducibility

The postal questionnaire score correlated with the score given by RW following clinical assessment at research clinic (*r* = 0.63). The postal score also correlated with PM's score which was carried out on the same day as RW's assessment (*r* = 0.62). RW's and PM's scores also correlated with each other (*r* = 0.5).

## 10. Usability

All 426 patients from all the different clinical settings answered all 10 questions of the SFAQ, with 72% completing the picture. The MFPDI, which was completed at research clinic was less thoroughly completed, with several patients omitting to answer one or more question. 15/139 (11%) patients completed under half of the MFPDI and so were not able to be reliably scored. 

The SMOG reading age of the SFAQ was 11, which means that it should be possible for patients with a lower level of literacy to comprehend and answer it. The MFPDI had a similar SMOG reading age but has twice as many questions with three choices for answers.

## 11. Discussion

We present a ten-point foot and ankle; patients administered questionnaire with diagrams for rapid screening in routine rheumatology outpatients. The low SMOG reading age and inclusion of diagrams take account of the significant functional illiteracy rate in the UK. It has been used in a number of different clinical settings and it has been completed by over 400 patients, with clinical correlation in half this group. It is feasible for patients to complete it prior to clinical review with a high score or picture indicating problems prompting a thorough foot history and examination. Completion of the diagrams could have resulted in correct diagnosis of foot or ankle problems in more than 70% of patients and can guide a more focused clinical assessment. We feel the SFAQ has a role as a useful practical adjunct in the global assessment of patients with RA, with a high score indicating the presence of clinically relevant foot pathology. It is not intended to act as a diagnostic tool.

Different domains of our questionnaire did not correlate with each other suggesting that they were each relatively independent variables. All questions were answered by all patients and therefore we felt that there were no redundant questions. Because most patients were retired, the question about work (question 5) was often amended by the patient if they did not work, but it was still answered. We felt it was important to retain this question to highlight the importance of work to patients and staff reviewing them. Similarly the questions about footwear were designed to prompt and educate both patient and staff about the importance of footwear in RA patients. 

Neither the SFAQ score nor the MFPDI correlated with other disease outcome measures used in the assessment of RA, suggesting that relying on these scores alone during a clinic appointment may miss important foot pathology. 

We have demonstrated that there is a reasonable level of correlation between the SFAQ and clinical assessment, and between the SFAQ and the MFPDI. 

The lack of correlation with the foot pain VAS was interesting. It highlights that pain is not the only important foot symptom limiting activities of daily living and affecting quality of life. 

The lack of correlation of the SFAQ or the MFPDI with HAQ and HAD scores contradicts findings from previous papers [3, 4]. Our patient group had a mean HAQ score of 1.2 compared to a mean score of 0.5 from the group from Rojas-Villarraga et al. [4] Wickman et al. did not use the HAQ and their patients had mild-moderate disease only. These differences may in part explain the differing results. 

There are currently a number of other available tools to assess foot symptoms. The MFPDI has been validated in a broad range of patient populations, including general rheumatology outpatients and older patients [20]. This led us to choose it as the comparator for our questionnaire. It has 20 questions, each with 3 options; our experience in clinic was that patients often found it difficult and tiring to complete. The Foot Health Status Questionnaire is very comprehensive and also validated in large patient numbers but has not been specifically validated for rheumatology patients [24]. The Foot Function Index is widely used but criticised by some for not assessing constructs such as footwear and participation restriction [25]. Of the 11 currently available patient-reported outcome measures (PROMs) used in the assessment of foot symptoms, only one is specific to Rheumatoid Arthritis—the LFIS [26]. We were not able to access the questionnaire as we were designing our own foot impact score. A revised LFIS was validated and compared with other outcome measures including the MFPDI and the HAQ in 135 patients by postal survey with good incremental progression between the different scores. A recent systemic literature review of the currently used PROMs to assess feet concluded that there was still a need to develop an RA foot-specific PROM [27].

Our patient group is typical of a district general hospital population with a high prevalence of foot problems, consistent with previous studies [1–7, 24–26]. Grondal et al. [28] found the most common area affected was the forefoot, but over 50% of patients also had hindfoot or ankle pathology. This finding was replicated in our study and highlights the importance of including the ankle as part of the foot assessment in RA. Jaakkola and Mann [29] found that 40% of their patients reported ankle pathology as more problematic than forefoot pathology. Those patients with initial forefoot involvement were in a poorer prognostic category [30]. As in our cohort, other studies have confirmed high rates of nonarticular foot pathologies, including posterior tibial tendinitis, hindfoot valgus, pes planus, tarsal tunnel, and neuromas. 

In many hospitals, access to timely podiatry review is limited. Early identification of foot and ankle problems using this questionnaire may act as prompt to timely interventions such as foot orthotics, which have been shown to reduce longer term permanent foot disability [31, 32]. Conversely, highlighting the high prevalence of foot pathology and the disability caused by RA may be a means of improving clinical services. 

The SFAQ is not intended to replace more detailed foot scoring systems such as the MFPDI, Foot Health Status Questionnaire, Foot Function Index, or the LFIS, or to be used as part of a more detailed podiatric assessment. The questionnaire was designed primarily for RA follow-up patients but can usefully be extended to other inflammatory arthritides. The questionnaire is simple enough to be completed unaided prior to the consultation. The inclusion of the diagrams is unique and a useful aid to patients to help them explain problems, and for clinicians to aid diagnosis. We feel this questionnaire has a useful role as a patient reported foot symptom tool for rapid screening of foot pathology during routine rheumatology review. 

The SFAQ is available to download.

## Figures and Tables

**Table 1:** Clinical scores from research clinic.

Score (possible range)	Range	Median	Mean	
Postal foot score SFAQ (0–10)	0–9	4	4	
Registrar clinical score (0–10)	0–10	4	4.1	
Podiatry score (0–10)	0–10	5	4.7	
Manchester score (0–40)	0–40	16	16	
DAS (1.1–9.31)	1.1–6.19	3.04	3.09	
HAQ (0–3)	0–3	1.25	1.21	
OSRA activity (0–10)	0–6	0	1	
OSRA damage (0–10)	0–8	1	1.5	

				Number (%) ≥ 11

HAD depression (0–21, ≥11 significant)	0–17	10	8.9	46 (36)
HAD anxiety (0–21, ≥11 significant)	0–19	8	7.9	41 (32)

**Table 2:** Interventions from research clinic.

Intervention	Number of patients (%)	Foot questionnaire score Median (range)	Clinic score Median (range)
None	74 (55)	3 (0–9)	3 (0–9)
Injection	10 (7)	4 (1–7)	5 (1–7)
Orthotics	39 (29)	6 (0–9)	4 (0–10)
Surgical referral	16 (12)	4 (0–7)	4 (2–9)
More than 1	9 (7)	5 (1–8)	5 (0–7)

**Table 3:** Diagnoses from research clinic.

Diagnosis	Number of patients			% patients		
Hallux valgus	56			41		
Callous	27			20		
MTP synovitis	25			18		
Ankle varus	22			16		
Claw toes	21			15		
Moretons neuroma	20			15		
Posterior tibial tendonitis	16			12		
Peroneal tendonitis	16			12		
Hind foot pathology	15			11		
Ankle pathology	14			10		
Hammer toes	14			10		
Other	10			7		
Previous surgery	5			4		
Cross over	3			2		
Achilles tendonitis	1			1		
Plantar fasciitis	2			1		

Number of diagnoses (%)	0	1	2	3	4	≥5

	12 (9)	35 (26)	58 (42)	21 (15)	10 (7)	2 (1)

**Table 4:** Diagnoses from general rheumatology and podiatric clinic.

Diagnosis	Rheumatology clinic Number (%) patients	Podiatric clinic Number (%) patients
MTP synovitis	8 (12)	18 (43)
Hallux valgus	8 (12)	5 (12)
Callous	6 (9)	3 (7)
Ankle pain	6 (9)	
Posterior tibialis dysfunction	4 (6)	5 (12)
Achilles tendinitis	3 (5)	
Dropped arch	3 (5)	
MTP subluxation	3 (5)	
Plantar fasciitis	3 (5)	
Claw toe	2 (3)	1 (2)
Hammer toe	2 (3)	
Moretons neuroma	1 (2)	9 (21)
Peripheral oedema	1 (2)	
Ulcer	1 (2)	
Subtalar		2 (5)
Bursa		2 (5)
Cross over toe		1 (2)

**Table 5:** Sensitivity and specificity thresholds of SFAQ.

Score	% patients needing new intervention	Score	% patients needing new intervention	Threshold	Sensitivity	Specificity	Positive predictive value	Negative predictive value
0	38%	≥1	54%	≥1	0.97	0.18	0.48	0.88
≤1	45%	≥2	48%	≥2	0.87	0.28	0.48	0.73
≤2	40%	≥3	51%	≥3	0.77	0.41	0.51	0.7
≤3	31%	≥4	54%	≥4	0.69	0.54	0.69	0.54
≤4	35%	≥5	55%	≥5	0.57	0.63	0.55	0.66
≤5	37%	≥6	57%	≥6	0.44	0.74	0.57	0.63
≤6	39%	≥7	63%	≥7	0.31	0.86	0.63	0.61
≤7	42%	≥8	70%	≥8	0.11	0.96	0.7	0.58
